# *In vitro* and *in vivo* activity of quisinostat against *Toxoplasma gondii*

**DOI:** 10.1128/aac.01819-24

**Published:** 2025-07-31

**Authors:** Hui-Jie Qiu, Wen-Bin Zheng, Ting Zeng, Shu-Feng Yang, Dai-Ang Liu, Li-Yan Wang, Zhi-Rong Liu, Xing-Quan Zhu, Chun-Xue Zhou

**Affiliations:** 1 Laboratory of Parasitic Diseases, College of Veterinary Medicine, Shanxi Agricultural University, Taigu, Shanxi, China; 2 Department of Pathogen Biology, School of Basic Medical Sciences, Cheeloo College of Medicine, Shandong University 66555 , Jinan, Shandong, China; 3 Shandong Public Health Clinical Center, Cheeloo College of Medicine, Shandong University 66555 , Jinan, Shandong, China; 4 National Institute of Parasitic Diseases, Chinese Center for Disease Control and Prevention (Chinese Center for Tropical Diseases Research), NHC Key Laboratory of Parasite and Vector Biology, WHO Collaborating Center for Tropical Diseases, National Center for International Research on Tropical Diseases, Shanghai, China; The Children's Hospital of Philadelphia, Philadelphia, Pennsylvania, USA

**Keywords:** *Toxoplasma gondii*, quisinostat, EC_50_, proteome, mouse

## Abstract

*Toxoplasma gondii,* an opportunistic pathogen, poses severe threats to immunocompromised individuals and fetuses of newly infected pregnant women. The current gold-standard treatment, a combination of pyrimethamine and sulfadiazine, is limited by severe adverse events, necessitating the development of novel therapeutic agents. *In vitro*, a CCK8 assay demonstrated that quisinostat inhibited HeLa cell proliferation in a dose-dependent manner, with a CC_50_ of 8.22 nM. Regarding *T. gondii* tachyzoites, quisinostat exhibited time-dependent inhibition of extracellular parasite activity and suppressed intracellular parasite proliferation, with an EC_50_ of 25.84 pM, and a high selectivity index (SI = 318.11). Quisinostat disrupted the *T. gondii* lytic cycle by decreasing invasion rates, inducing G1 cell cycle arrest, reducing replication, and shrinking plaque size. Ultrastructural analysis indicated that quisinostat treatment led to membrane damage, enhanced lactate dehydrogenase (LDH) release, and apoptotic cell death in tachyzoites, whereas no significant change in reactive oxygen species (ROS) levels was detected. Proteome analysis identified 77 upregulated and 205 downregulated proteins, which were enriched in functions associated with protein dephosphorylation and ion transport, as well as pathways, such as non-homologous end-joining. Molecular docking studies revealed a strong interaction between quisinostat and *T. gondii* HDAC3. *In vivo*, treatment with quisinostat increased the survival time of mice infected with virulent RH strain. In mice infected with low-virulent ME49 tachyzoites, quisinostat treatment decreased parasite burden in multiple organs and increased the survival to 80%. Taken together, these findings demonstrate that quisinostat has potent anti-*Toxoplasma* activity both *in vitro* and *in vivo*, which offers promise for treatment of human toxoplasmosis.

## INTRODUCTION

Toxoplasmosis is one of the most important zoonotic parasitic diseases, caused by the obligate intracellular protozoan *Toxoplasma gondii*, which can infect almost all warm-blooded animals, including humans (1, 2). Globally, approximately one-third of the world’s human population is exposed to or has latent infection with toxoplasmosis (3, 4). Most human and animal infections caused by *T. gondii* occur after birth through the consumption of raw or undercooked meat containing *Toxoplasma* cysts, or through the ingestion of fruits, vegetables, or water contaminated with *Toxoplasma* oocysts (5). Although *T. gondii* infection is usually asymptomatic in immunocompetent individuals, it can lead to serious complications in individuals with compromised immune systems. Moreover, infections during pregnancy may result in miscarriage, stillbirth, or severe congenital defects, such as blindness, intellectual disability, and hydrocephalus (1, 2). Additionally, studies have shown that latent infection is associated with a broad spectrum of neuropsychiatric and behavioral disorders (6).

Traditional anti-toxoplasmosis drugs include sulfonamides, macrolides, and quinolines. The combination of sulfadiazine and pyrimethamine is the most effective therapy for toxoplasmosis in clinical practice. They mainly target dihydrofolate reductase and dihydrofolate synthetase, thereby blocking folate synthesis and achieving anti-*Toxoplasma* effects (7). However, this combination sometimes causes some serious adverse effects, such as bone marrow toxicity and skin rash (8). Macrolides are a class of drugs used to treat infections caused by Gram-positive bacteria. Members of antibiotic macrolides also have good clinical effects on toxoplasmosis. For example, spiramycin is recommended to treat toxoplasmosis in early pregnancy, which remarkably reduces the risk of transferring the disease to the fetus (9). Other drugs such as azithromycin, clarithromycin, and aminophylline are effective drugs against toxoplasmosis (10). However, these drugs are mainly effective in the acute phase of infection and are unable to eradicate encysted parasites in the tissues. Thus, novel therapeutic strategies are urgently needed, particularly for targeting chronic infection stages. To discover more effective drugs, many compounds targeting epigenetic mechanisms have been tested and demonstrated significant anti-protozoan activity. Several studies have shown that the enzymes mediating epigenetic modifications of histones have been verified as one of the promising therapeutic targets (11, 12). In fact, the regulation of gene expression is commonly found throughout the life cycle of parasites and is of vital importance for their reproduction and survival. Histone acetylation, a well-studied epigenetic mechanism in tumor pathogenesis, has recently garnered attention for its potential in identifying compounds capable of inhibiting parasite activity (13–15). The enzymes involved in the regulation of histone acetylation are crucial for the proliferation and pathogenesis of *T. gondii* (16). Moreover, these enzymes have been determined as promising therapeutic targets since a variety of regulators targeting these enzymes have been proved to have inhibitory effects on *T. gondii*. For instance, the hydroxamate-based HDAC inhibitor (HDACi) MC1742 can prevent the consequences of acute toxoplasmosis in mouse models, and it also has inhibitory effects on other apicomplexan parasites (17). FR235222 and its derivatives show high selectivity and HDAC6 inhibitory activity and have a dramatic effect on *T. gondii* tachyzoite growth (18). These findings underscore a crucial role of histone deacetylation in maintaining the specific gene expression program during the life cycle of *T. gondii*.

Quisinostat is a hydroxamate-based HDACi, demonstrating broad-spectrum antiproliferative activity against multiple cancer cells (19). It can strongly inhibit Class I and Class II HDACs and has displayed sustained H3 acetylation and effective anti-tumor activity in preclinical *in vivo* models of human cancers (20). Additionally, quisinostat has exhibited robust anti-tumor activity in mouse multiple myeloma models, with almost complete alleviation of tumor burden and significant reduction in angiogenesis (21). Previous investigations indicate that quisinostat and its derivatives have remarkable anti-*malarial* effects (22). Nevertheless, it remains unclear whether quisinostat possesses anti-*Toxoplasma* effects. In the present study, we evaluated the activity of quisinostat against *T. gondii* both *in vitro* and *in vivo* and preliminarily explored its mechanism of action in anti-*T*. *gondii* activity.

## MATERIALS AND METHODS

### Animals and parasites

Female C57BL/6 and BALB/c mice aged 6-8 weeks were obtained from the Laboratory Animal Center of Shandong University, China. All mice were acclimated for 1 week prior to the study and had free access to sterilized water and food. Throughout the study, certified animal care experts monitored the overall health of the mice regularly.

*T. gondii* RH strain (type I) and ME49 strain (type II) were grown in confluent monolayers of human foreskin fibroblast (HFF) cells (HS27; ATCC: CRL-1634) maintained in Dulbecco’s Modified Eagle Medium (DMEM) (Cellmax, China) supplemented with 2% fetal bovine serum (FBS) (Cellmax, China), as well as 2 mM glutamine, 100 U/ml of penicillin, and 10 µg/ml of streptomycin (Servicebio, China) at 37°C, 5% CO_2_.

### Compounds

Quisinostat was purchased from MedChem Express (MCE), and dissolved in dimethyl sulfoxide (DMSO) (CAS: 67-68-5, Solarbio, China), and kept at −80°C until use. Pyrimethamine was purchased from Sigma-Aldrich (US CAS: 58-14-0).

### *In vitro* cytotoxicity study

The cell viability was assessed using the Cell Counting Kit-8 (CCK-8, TargetMol, USA) as previously described (23). Briefly, HeLa cells were cultured in 96-well plates at a density of 5 × 10^3^ cells per well and then incubated for 12 h. After washing with phosphate buffer solution (PBS, pH 7.4), the cells were incubated with fresh complete medium containing quisinostat at different concentrations (0.06, 0.12, 0.24, 0.49, 0.97, 1.95, 3.91, 7.81, 15.63, 31.25, and 62.50 nM) for 48 h. Cell Counting Kit-8 solution was then added to each well and incubated at 37  °C for 2 h. To evaluate cell viability, the optical density at 450  nm was measured using a microplate reader (ELx800, BioTek Inc., USA).

### Parasite quantification

Parasite replication was measured by absolute quantitative PCR method as previously described (24). Briefly, genomic DNA (gDNA) was extracted from all samples, and then the amplification of the *T. gondii* B1 gene was performed with parasite-specific primers (5′-TGAGTATCTGTGCAACTTTGG-3′and 5′-TCTCTGTGTACCTCTTCTCG-3′). The gDNA was added to a 2 × Taq PCR MasterMix (Tiangen, Beijing, China) containing 10 pM/L of the primer pairs. Amplification was performed on Bio-Rad CFX Connect Real-Time PCR Detection System (Bio-Rad Laboratories, Inc.) with 40 cycles of denaturation at 95°C for 10 s, annealing at 60°C for 32 s and extension. A standard curve was generated using 10-fold serial dilutions of gDNA extracted from 10^7^ parasites and was used to compute the parasite number in the test samples.

### Determination of half-maximal effective concentration (EC_50_) against *T. gondii*

EC_50_ assay was performed as previously described (25). Fresh egressed *T. gondii* RH-GFP tachyzoites (MOI 3) were inoculated onto HeLa cells for 4 h. Following the removal of extracellular *T. gondii* parasites, host cells were incubated with complete culture medium supplemented with varying concentrations of drugs. Culture medium containing 0.1% DMSO (vehicle) was used as negative control. After incubation for 48 h, fluorescence images were obtained using Zeiss Axio Vert. A1 microscope (Carl Zeiss AG, Germany) with a 20  ×  objective using ZEN imaging software (Carl Zeiss). The fluorescence areas of RH-GFP were calculated by Image J software.

### *In vitro* invasion assay

A two-color staining protocol was used to determine the invasion efficiency as previously described (26). Purified tachyzoites of *T. gondii* RH strain (MOI 1) were inoculated onto HeLa cells for 4 h in medium containing quisinostat (50 pM) or 0.1% DMSO (vehicle). Supernatant was removed, and cells were fixed with 4% paraformaldehyde (PFA, Biosharp, BL539A, China) for 20 min. Parasites attached to HeLa cells were labeled with mouse anti-SAG2 polyclonal antibody (prepared in our laboratory) diluted at 1:500 and Alexa Fluor 594-conjugated goat anti-mouse IgG (Beyotime, A0521, China). Cells were then permeabilized with 0.1% Triton X-100 for 15 min and blocked with 10% FBS. Parasites were labeled using anti-IMC1 antibodies (prepared in our laboratory), and then stained with Alexa Fluor 488-conjugated goat anti-mouse IgG (Invitrogen, A-11001, USA). The nuclei were detected by DAPI staining (Solarbio, China, CAS: 28718-90-3). At least 10 visual fields were randomly selected for each slide to count the number of host nucleus, extracellular parasites, and the whole parasites. The invasion rate was calculated as: Invasion Rate (%) = [(Total Parasites - Extracellular Parasites) / Total Parasites] ×100.

### *In vitro* replication assay

To investigate the intracellular replication efficiency of the parasite treated with quisinostat, HeLa cells grown in 24-well plates were infected with 5 × 10^5^ *T. gondii* RH strain (MOI 1) per well for 4 h. After removing the extracellular parasites by washing with PBS, cells were treated with quisinostat (50 pM) or 0.1% DMSO (vehicle) for 24 h. Tachyzoites within vacuoles were labeled with mouse anti-IMC1 and counted in at least 100 vacuoles per slide.

### Plaque assays

*In vitro* plaque assays were performed on HFF cells cultured as previously described (27). Approximately 200 freshly egressed RH tachyzoites were inoculated onto monolayers of HFF cells in a 12-well plate and grown for 9 days in medium containing quisinostat (25 pM) or 0.1% DMSO (vehicle). Cells in the mock group were not infected with parasites and cultured in medium containing 0.1% DMSO. The cells were washed three times with PBS and then fixed with 4% PFA (Biosharp, BL539A, China) for 20 min and then stained with 0.2% crystal violet for 20 min. The plaques were imaged and quantified using the Image J software.

### Analysis of cell cycle

The impact of quisinostat on the cell cycle of *T. gondii* tachyzoites was evaluated using the Cell Cycle and Apoptosis Assay Kit (Beyotime, C1052, China) as previously described (28). The purified tachyzoites were added into HeLa cells (MOI 5) and incubated for 4 h. Extracellular parasites were removed, and cells were cultured in medium containing quisinostat (50 pM) or DMSO (vehicle) for 24 h. Parasites were released from infected host cells by passage through a 27-gage needle followed by filtration using a 3 µm polycarbonate membrane filter. Purified tachyzoites were fixed with ice-cold 70% ethanol for 2 h and then stained with propidium iodide (PI). The cell cycle was monitored on a Cytoflex flow cytometer (Beckman Coulter, Brea, CA) with 10,000 events per sample and analyzed with ModFit LT software (Verity, Topsham, ME).

### Transmission electron microscopy analysis

Transmission electron microscopy (TEM) analysis was performed as previously described (29). Briefly, fresh egressed tachyzoites of RH strain were treated with quisinostat (50 pM) or DMSO (vehicle) for 12 h. The parasites were fixed with 2.5% glutaraldehyde in phosphate buffer (0.1 M) followed by post-fixation with 1% osmium tetroxide. The sample was dehydrated with acetone and embedded in resin, followed by cutting of thin sections with an ultramicrotome. Sections were observed under transmission electron microscopy (EM10C-100 KV; Carl Zeiss, Germany).

### LDH assay

Lactate dehydrogenase (LDH) was detected as previously described (30). Briefly, purified tachyzoites of *T. gondii* RH strain were treated with quisinostat (50 pM) or 0.1% DMSO (vehicle) for 24 h. The levels of LDH were detected using the LDH Cytotoxicity Assay Kit (Beyotime, China) according to the manufacturer’s instructions.

### ROS assay

Reactive oxygen species (ROS) were detected using the ROS assay kit (Beyotime, S0033S, China) as previously described (31). Briefly, RH tachyzoites were treated with quisinostat (50 pM) or DMSO (vehicle) for 2, 6, or 12 h. The parasites were collected, washed with pre-cooled PBS, and stained with 10 µM DCFH-DA at 37℃ in the dark for 20 min, which was then detected by Cytoflex flow cytometer (Beckman Coulter, Brea, CA).

### Apoptosis analysis

Apoptosis detection was performed by using Annexin V-FITC Apoptosis Detection Kit (Beyotime, China) as previously described (32). Fresh egressed tachyzoites of *T. gondii* RH strain were treated with quisinostat (50 pM) or 0.1% DMSO (vehicle) for 24 h. The parasites were collected and re-suspended with 195 µL binding buffer and incubated in darkness at room temperature with 5 µL Annexin V-FITC and 10 µL PI for 15 min. The samples were analyzed by Cytoflex flow cytometer (Beckman Coulter, Brea, CA), and the results were presented as percentage of apoptotic cells.

### Proteomic analysis

Fresh egressed tachyzoites of RH strain were collected and treated with quisinostat (50 pM) or DMSO (vehicle) for 12 h. The samples were collected and lysed by adding an appropriate amount of DB protein lysis buffer (6 M urea, 100 mM TEAB, pH 8.5), followed by vortexing and sonication in an ice-water bath for 5 min. After centrifugation at 12,000 g for 15 min at 4°C, the supernatant was collected and incubated with 1 M DTT at 56°C for 1 h. Following a 2 min ice bath, the sample was alkylated with iodoacetamide (IAM) in the dark at room temperature for 1 h. Protein concentration was determined using the Bradford assay, with BSA standards ranging from 0–0.5 µg/µL. Samples and standards (20 µL) were added to a 96-well plate in triplicate, mixed with 180 µL of G250 reagent, incubated for 5 min at room temperature, and absorbance was measured at 595 nm. Protein samples (20 µg) were separated by 12% SDS-PAGE (stacking gel: 120 V, 20 min; resolving gel: 150 V, 50 min) and visualized by Coomassie Brilliant Blue R-250 staining. For proteolytic digestion, samples were adjusted to 100 µL with DB lysis buffer, incubated with trypsin and 100 mM TEAB at 37°C for 4 h, acidified with formic acid (pH <3), and centrifuged at 12,000 g for 5 min. Peptides were desalted using a C18 column, washed thrice with 0.1% formic acid/3% acetonitrile, eluted with 0.1% formic acid/70% acetonitrile, and lyophilized.

Proteomic sequencing services were provided by Novogene Co., Ltd. (Tianjin, China). Samples were analyzed using a Vanquish Neo ultra-high performance liquid chromatography (UHPLC) system (Thermo Scientific) coupled with an Orbitrap Astral mass spectrometer (Thermo Scientific) for LC-MS/MS analysis. Protein detection was performed using data-independent acquisition (DIA) technology. Raw data files were processed with DIA-NN software (Version 1.8.1) against the UniProt database (*T. gondii* RH strain, UniProtKB entry 8552). The mass spectrometry proteomics data have been deposited to the ProteomeXchange Consortium (https://proteomecentral.proteomexchange.org) via the iProX partner repository with the data set identifier PXD064903. False discovery rate (FDR) validation was employed to filter out peptides and proteins with an FDR > 1%. Differential abundance proteins (DAPs) were identified based on the following criteria: two-sample *t*-test *P*-value < 0.05 and fold change (FC) ≥1.5 or ≤0.67.

Functional annotation of identified proteins was performed using InterProScan software (v105.0, European Bioinformatics Institute, Cambridge, UK), encompassing Gene Ontology (GO) and InterPro (IPR) annotations based on databases including Pfam, PRINTS, ProDom, SMART, ProSite, and PANTHER. Differential expression analysis was conducted using volcano plots, hierarchical clustering heatmaps, and enrichment analyzes of GO terms and Kyoto Encyclopedia of Genes and Genomes (KEGG) pathways.

#### Molecular docking

Due to the absence of an experimentally determined structure for *T. gondii* RH strain HDAC3 (UniProt ID: Q2Y2R0), the full-length amino acid sequence (451 residues) was retrieved from UniProt. The 3D structure of HDAC3 was then predicted using AlphaFold2 and accessed from the AlphaFold Protein Structure Database (https://alphafold.ebi.ac.uk/). For the ligand preparation, the 3D structure of Quisinostat (PubChem CID: 11538455) was downloaded in SDF format and converted to MOL2 using OpenBabel v3.1.1. Both the ligand and protein structures were optimized using PyMOL v2.3.3, with the ligand undergoing geometry cleaning and the protein structure refined by adding explicit hydrogens, removing crystallographic water molecules and non-essential ions, and adjusting protonation states of ionizable residues. Molecular docking simulations were performed using AutoDock Vina v1.2.5 (https://vina.scripps.edu). Docking results were analyzed and visualized in PyMOL 3.12, focusing on hydrogen bonds, hydrophobic interactions, and key residue contacts, with the binding pose exhibiting the lowest free energy (ΔG) selected for further analysis.

### Anti-*Toxoplasma* activity *in vivo*

The *in vivo* anti-*T*. *gondii* efficacy of quisinostat was evaluated using BALB/c and C57BL/6 mouse models. Female BALB/c mice were intraperitoneally injected with 1000 fresh egressed RH tachyzoites. C57BL/6 female mice were intraperitoneally injected with 100 RH strain tachyzoites or 200 ME49 strain tachyzoites. One day after infection, the infected mice were randomly assigned to administration with vehicle (10% DMSO, 40% PEG-300, 5% Tween 80, and saline, i.p.) or quisinostat (10 mg/kg in 10% DMSO, 40% PEG-300, 5% Tween 80, and saline, i.p.) every two days for a total of 10 days. Mock group mice were intraperitoneally injected with sterile PBS instead of tachyzoites and then treated with quisinostat (10 mg/kg in 10% DMSO, 40% PEG-300, 5% Tween 80, and saline, i.p.) as above. The mice were euthanized by CO_2_ inhalation when a humane endpoint was reached or at the end of the study period.

### Statistical analysis

All statistical analyzes were performed using GraphPad Prism 7.0 software (GraphPad). All results were expressed as mean ± standard deviation (SD). Statistical differences were calculated using Student′s *t* test (two-tailed, unpaired), one-way ANOVA or two-way ANOVA, with a *P* value < 0.05 considered statistically significant.

## RESULTS

### Quisinostat inhibited *T. gondii* growth *in vitro*

**Fig 1:**
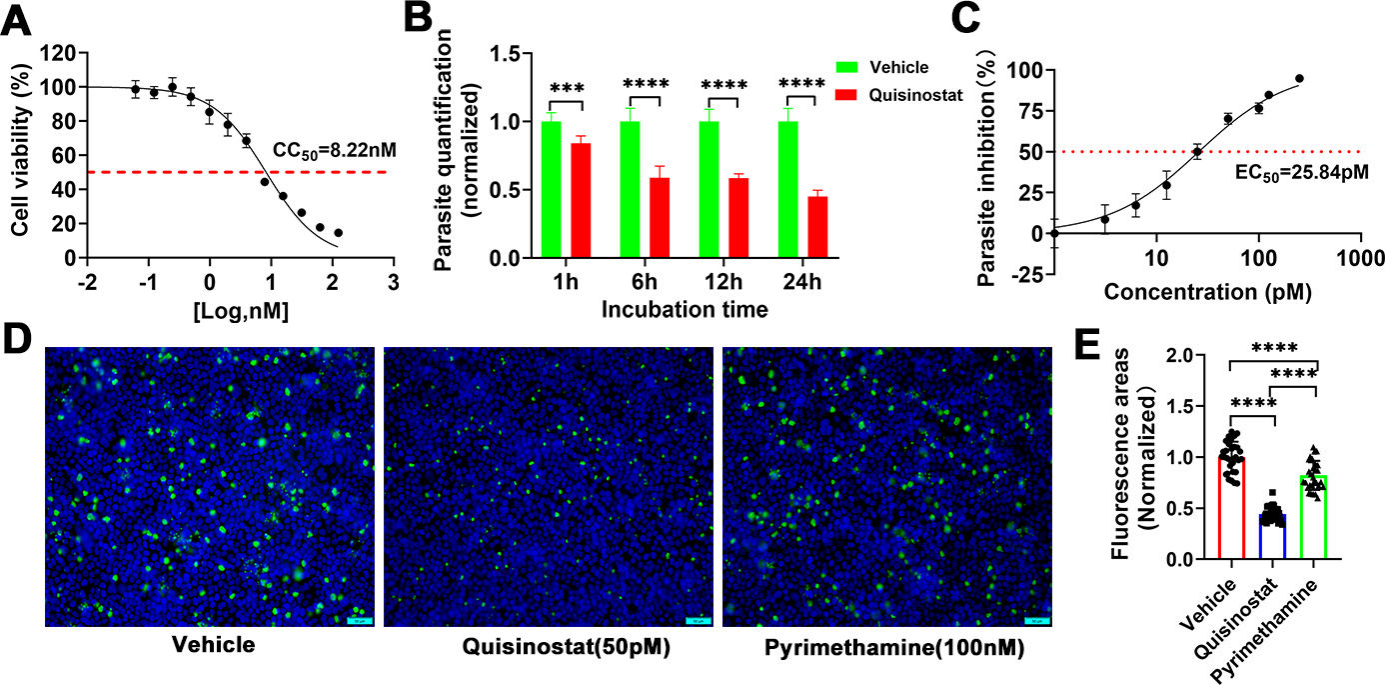
*In vitro* activities of quisinostat. (**A**) *In vitro* toxicity of quisinostat to HeLa cells. CC_50_ was determined by CCK8 assays. (**B**) Effects of quisinostat on the activity of extracellular *T. gondii* RH tachyzoites. Fresh purified RH tachyzoites were pretreated with quisinostat (50 pM) or vehicle (0.1%) DMSO for 1, 6, 12, or 24 h, and then inoculated onto HeLa cells (MOI 1), and cultured for 24 h. Samples were collected for quantification. The *P* values by two-way ANOVA are indicated; ***P < 0.001 and ****P < 0.0001. (**C**) Dose response curves of *T. gondii* RH-GFP tachyzoites exposed to different concentrations of quisinostat. EC_50_ was determined. (**D**) Indirect immunofluorescence assay demonstrated inhibitory effects of quisinostat on RH tachyzoites. Host cells infected with RH strain tachyzoites were treated with quisinostat (50 pM), pyrimethamine (100 nM), or 0.1% DMSO (vehicle) for 24 h. The cells were stained with DAPI and mouse anti-IMC1 antibody. Representative immunofluorescence images of parasites treated with vehicle (0.1% DMSO) and quisinostat are shown. (**E**) Quantitative analysis of fluorescence intensity for (**D**). Normalized values represent means ± SD, 10 fields from each of 3 independent experiments. The *P* values by one-way ANOVA are indicated are indicated; ****P < 0.0001.

The *in vitro* cytotoxicity of quisinostat on cell proliferation was evaluated using a CCK8 assay in the HeLa cell line. Following a 48 hour incubation with various concentrations of quisinostat, we observed a significant, dose-dependent suppression of cell proliferation. The half-maximal cytotoxic concentration (CC_50_) of quisinostat in HeLa cells was determined to be 8.22 nM (Fig. 1A). Subsequently, the antiparasitic activity of quisinostat against *T. gondii* tachyzoites was investigated *in vitro*. Freshly egressed tachyzoites were pre-treated with quisinostat prior to infection. As depicted in Fig. 1B, quisinostat significantly inhibited the activity of extracellular parasites in a time-dependent manner. To assess its effect on intracellular parasites, we employed the *T. gondii* RH-GFP strain, which constitutively expresses green fluorescent protein (GFP). Quisinostat was added to confluent HeLa cells 4 hours post-infection. The results, shown in Fig. 1C, revealed that quisinostat inhibited the proliferation of *T. gondii* RH-GFP tachyzoites with an EC_50_ value of 25.84 pM. In comparison, pyrimethamine, used as a positive control, exhibited an EC_50_ of 0.60 µM (Fig. S1). Furthermore, indirect immunofluorescence assays demonstrated that quisinostat significantly impaired the activity of *T. gondii* RH tachyzoites, outperforming both the vehicle control group and the pyrimethamine group (Fig. 1D and E). Notably, the selectivity index (SI = CC_50_/EC_50_) of quisinostat was calculated as 318.11, underscoring its high safety profile and potent anti-*T*. *gondii* activity.

### Quisinostat impaired *T. gondii* lytic cycle in *vitro*

**Fig 2:**
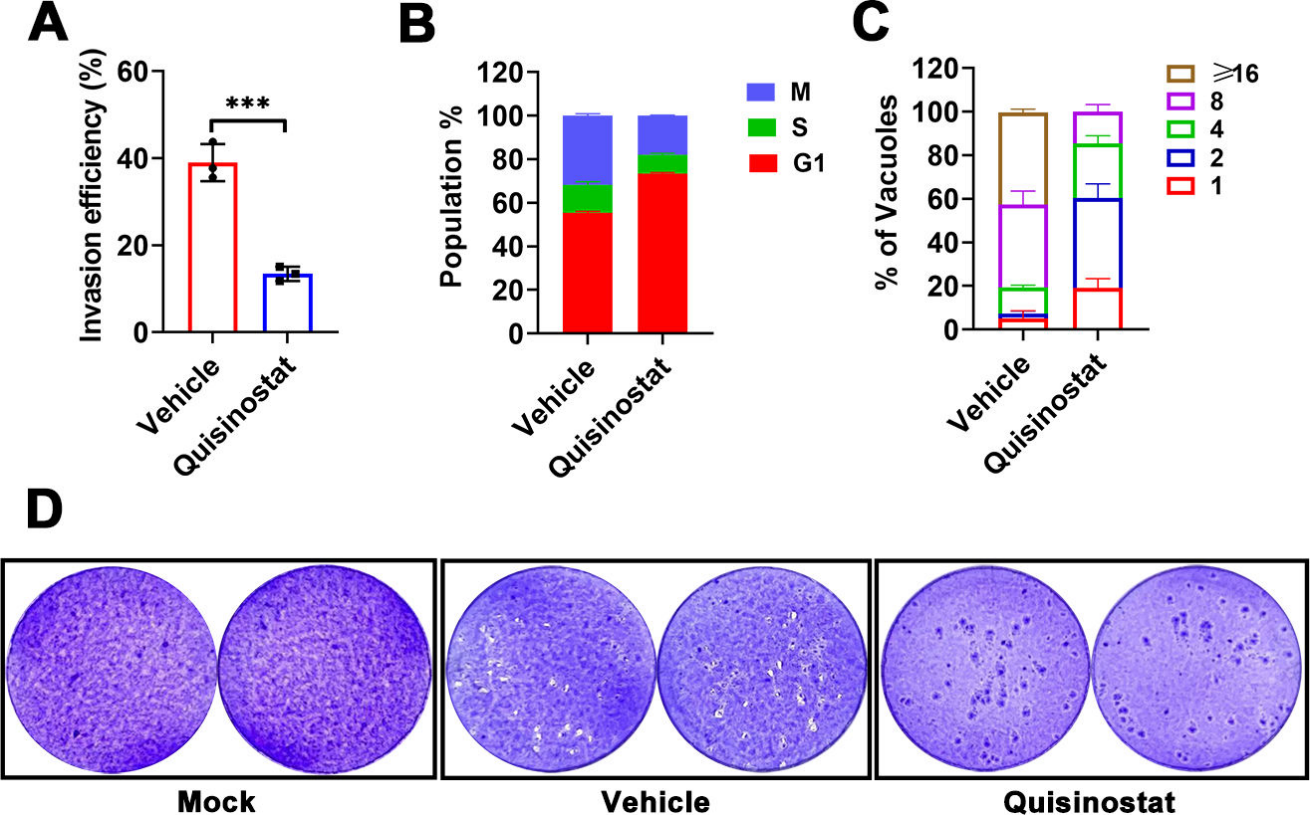
Quisinostat impaired *Toxoplasma gondii* lytic cycle *in vitro.* (**A**) Invasion assay to check the invasion capacity of RH tachyzoites treated with or without quisinostat. Invasion events were determined through two-color staining to discriminate invaded parasites from non-invaded ones, and results are presented as the percentages of invaded parasites for each group (*n*  =  3). The data presented represent the means ± SD derived from three independent experiments conducted in duplicate. The *P* values determined by Student′s *t*-test are indicated; ****P < 0.0001. (**B**) Cell cycle analysis of parasites treated with quisinostat or vehicle by using flow cytometry. These results represent the means ± SD from three independent experiments performed in triplicate. (**C**) Intracellular growth assay of RH tachyzoites upon 24 h growth with or without quisinostat. The numbers of PVs containing 2, 4, 8, or ≥16 parasites were counted by IFA. The numbers of parasites within 100 randomly selected vacuoles were counted. The data are presented as the means ± SD of at least three independent experiments. (**D**) Plaque assay of RH strain tachyzoites treated with or without quisinostat for 9 days.

We next investigated the effect of quisinostat on the entire lytic cycle of *T. gondii*. As shown in Fig. 2A, the invasion rate of parasites treated with quisinostat was significantly decreased compared to the vehicle group (*P* < 0.001). Subsequently, we performed cell cycle analysis of RH tachyzoites by flow cytometry to determine whether parasites treated with quisinostat were cell cycle arrested. For the drug-treated parasites, dramatic G1 cell cycle arrest (73.41% ±0.44% G1, 8.65% ± 0.57% S, 17.95%±% M) was observed, in contrast to the normal cell cycle profile of vehicle-treated cell population (55.42%±0.67% G1, 12.81% ± 1.45% S, 31.76%± 0.88% M) (Fig. 2B). An intracellular replication assay was used to examine the rate of parasite division following quisinostat treatment. As shown in Fig. 2C, the replication rate of drug-treated parasites was decreased compared to the vehicle group, since a notable reduction in the number of vacuoles containing eight or more parasites. We next performed plaque assays to test the effect of quisinostat on successive lytic cycles. Confluent monolayers of HFF cells were infected, and the parasites were allowed to replicate undisturbed for 9 days in medium containing quisinostat or not. As shown in Fig. 2D, the plaque number decreased and the plaque size was much smaller in the drug-treated group. These results indicate the exposure to quisinostat remarkably affects the ability of tachyzoites to efficiently progress through the lytic cycle *in vitro*.

### Quisinostat treatment induced ultrastructural changes, LDH release, and apoptotic cell death in *T. gondii* tachyzoites

**Fig 3:**
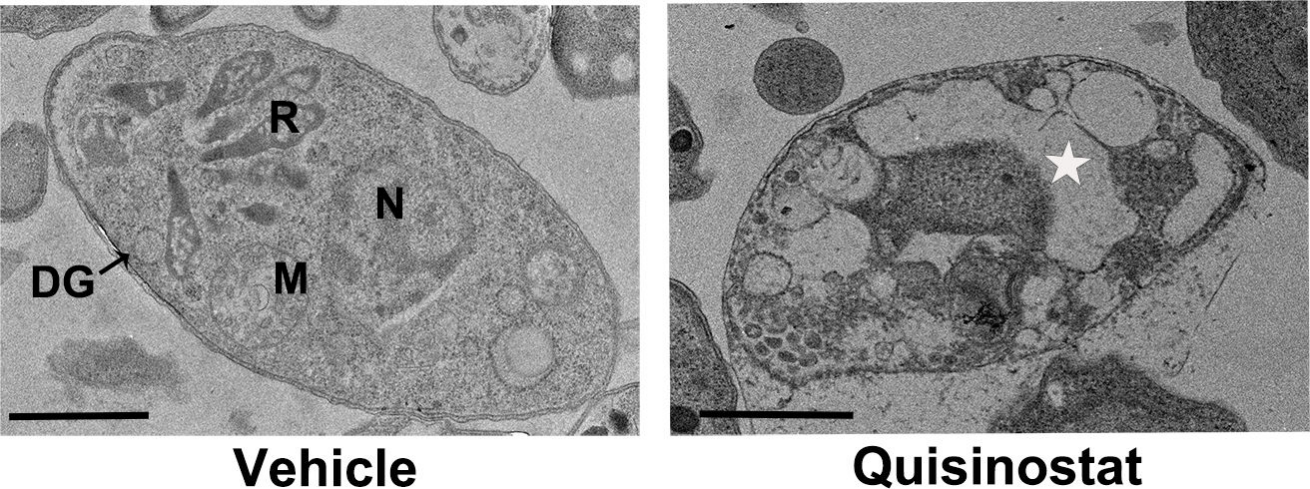
Ultrastructural changes in *T. gondii* tachyzoites after quisinostat treatment. Fresh purified RH tachyzoites were pretreated with quisinostat (50 pM) or vehicle (0.1%) DMSO for 12 h. In the vehicle group, well-preserved tachyzoite structures including rhoptries (R), dense granules (DGs), nuclei (N), and mitochondria (M). Incubation of quisnostat induced loss of cytoplasmic membrane integrity, and many autophagic vacuoles to emerge in the cytoplasm (☆). Scale bars: 1 µm.

**Fig 4:**
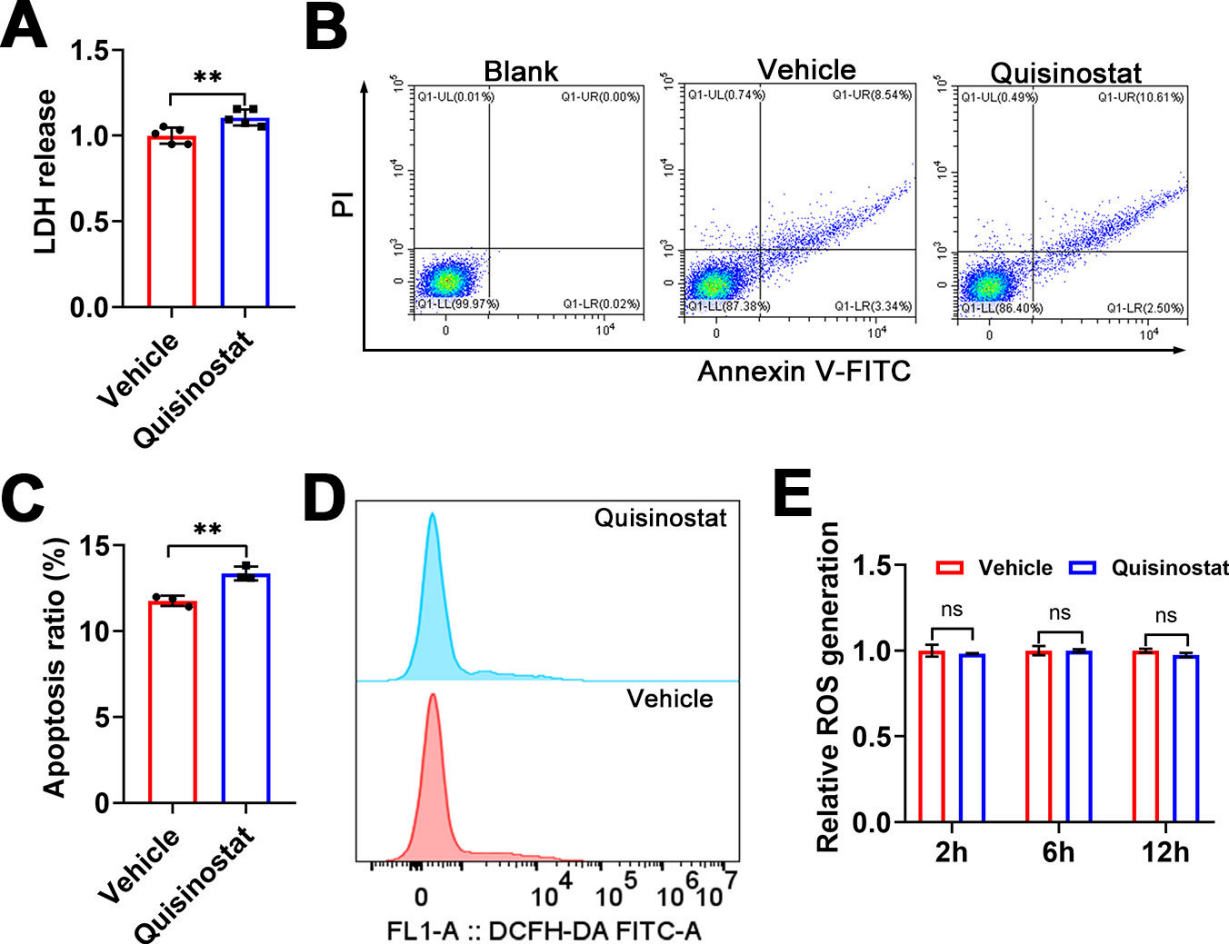
Quisinostat promoted LDH release and induced apoptosis in *Toxoplasma gondii*. (**A**) Effect of quisinostat on LDH release in RH tachyzoites. The *P* values by student’s *T* test are indicated; **P < 0.01. (**B**) Representative flow cytometric dot plots of annexin V-FITC/PI staining are shown. (**C**) Using Annexin V-FITC and PI staining to detect the percentage of apoptotic parasites treated with quisinostat or vehicle via flow cytometry. The mean percentages ± SD of annexin V^+^, PI^+^, or PI^-^ parasites were calculated from at least three independent experiments. The *P* values by Student′s *T* test are indicated; **P < 0.01. (**D**) DCFH-DA assay of the ROS generation in tachyzoites treated with quisinostat or 0.1% DMSO (vehicle) for 2 h. (**E**) Quantitative analysis of the intracellular ROS in tachyzoites treated with quisinostat or 0.1% DMSO (vehicle) for 2 h, 6 h or 12 h by flow cytometry. The results are presented as the means ± SD of at least three independent experiments. The *P* values by Student′s *T* test are indicated.

After 12 hour incubation with quisinostat, the RH tachyzoites exhibited notable ultrastructural abnormalities. As shown in Fig. 3, drug-treated parasites lost their cytoplasmic membrane integrity and were vacuolated without nuclei or organelles. While parasites in the vehicle group had intact plasma membranes, nuclei, mitochondria, rhoptries, and dense granules. LDH is a stable cytoplasmic enzyme that is rapidly released into the supernatant when the plasma membrane is damaged. As shown in Fig. 4A, incubation with 50 pM quisinostat significantly increased LDH release from RH tachyzoites, implicating pronounced cellular damage after exposure to quisinostat. As shown in Fig. 4B and C, by using flow cytometry analysis of Annexin V/PI staining, *T. gondii* tachyzoites exhibited significant increases in the percentage of apoptotic cells upon quisinostat treatment, which indicated that quisinostat-induced cellular damage may result from apoptotic cell death. A previous study showed that quisinostat kills cancer cells by augmenting ROS stress (33). However, there was no significant difference in ROS levels between drug-treated group and the vehicle group (Fig. 4D and E).

### Quisinostat altered the proteome of *T. gondii*

**Fig 5:**
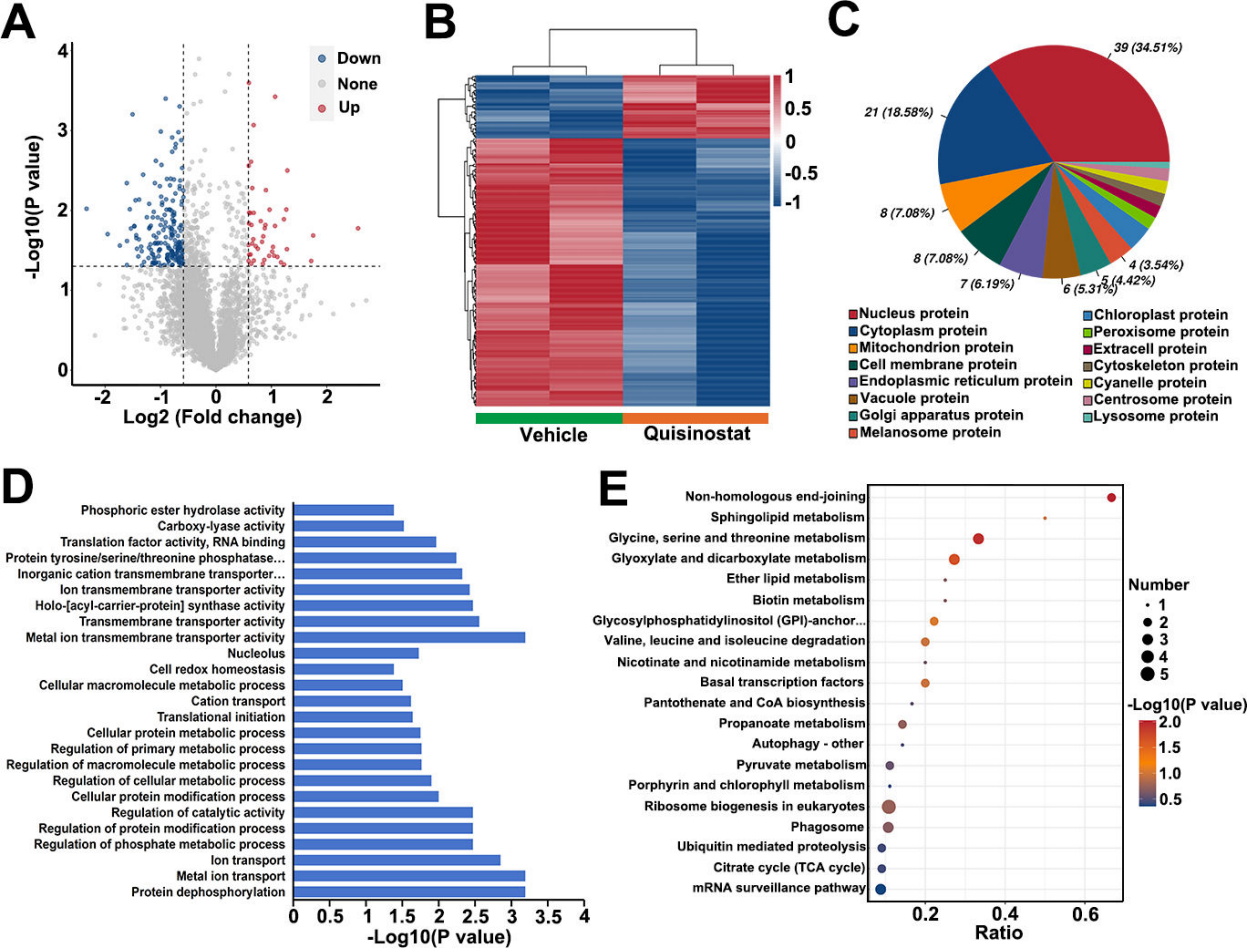
Proteomic analysis of *T. gondii* RH tachyzoites exposed to quisinostat. (**A**) Volcano plot depicting -log10 (*P* values) against protein expression fold-changes between the quisinostat-treated group and vehicle control (0.1% DMSO).(**B**) Unsupervised hierarchical clustering of proteomic data sets. Normalized protein abundance is color-coded: blue indicates low expression, while reddish hues denote high expression. Columns were clustered using complete linkage with Pearson correlation as the distance metric.(**C**) Subcellular localization analysis of differentially expressed proteins (DEPs) identified by mass spectrometry.(**D**) Gene Ontology (GO) enrichment analysis of DEPs. The top 25 significantly enriched GO terms are shown, with the x-axis representing -log10 (*P* values) and the y-axis denoting GO term categories.(**E**) KEGG pathway enrichment analysis of DEPs. The top 20 significantly enriched pathways are displayed, where the x-axis indicates the protein fold-change ratio, the y-axis lists KEGG pathway terms, dot color reflects -log10 (*P* values), and dot size corresponds to the number of DEPs per pathway.

To systematically explore the impact of quisinostat on the proteomic profile of *T. gondii*, a comprehensive proteome analysis was conducted. *T. gondii* RH tachyzoites were treated with 50 pM quisinostat or 0.1% DMSO (vehicle control) for 12 hours. Volcano plot analysis of differentially expressed proteins (DEPs) revealed 77 up-regulated and 205 down-regulated proteins when comparing the quisinostat-treated group to the vehicle group (Fig. 5A). The heatmap in Fig. 5B illustrates the proteomic distinctions between the two groups. Subcellular localization analysis of 113 identified DEPs showed predominant distributions in nuclear proteins (34.51%), cytoplasmic proteins (18.58%), and mitochondrial proteins (7.08%) (Fig. 5C). To characterize DEP functions, Gene Ontology (GO) enrichment analysis was performed. The top 25 enriched GO terms (Fig. 5D) were led by “Protein dephosphorylation”, “Metal ion transport”, and “Ion transport”. Pathway enrichment analysis using KEGG further identified the top three significantly enriched pathways: “Non-homologous end-joining”, “Glycine/Serine/Threonine metabolism”, and “Glyoxylate/Dicarboxylate metabolism” (Fig. 5E).

**Fig 6:**
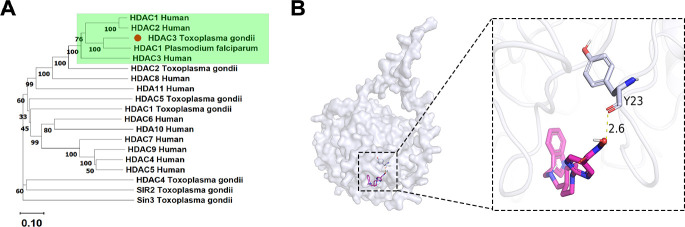
Characterization of small molecule binding sites in HDAC3. (**A**) Phylogenetic analysis of HDAC3 homologs across taxonomically representative organisms. Full-length protein sequences were retrieved from the National Center for Biotechnology Information (NCBI) database, and a maximum-likelihood phylogenetic tree was reconstructed using MEGA12 with 1,000 bootstrap replicates. (**B**) Cartoon representation of quisinostat docking to the active site of *T. gondii* RH strain HDAC3 (UniProt ID: Q2Y2R0). Quisinostat forms a hydrogen bond with the Tyr23 residue of HDAC3, as indicated by the dashed line.

Notably, the treatment of *T. gondii* with quisinostat—a pan-HDAC inhibitor that targets all human histone deacetylases (HDACs) and exhibits high potency against HDAC1, HDAC2, and HDAC3 (34)—resulted in a significant downregulation of HDAC2, HDAC3, and SIR2 expression (Table S1). Previous studies have established PfHDAC1 as the antimalarial target of quisinostat, suggesting its broader efficacy against apicomplexan parasites. Phylogenetic analysis confirmed a close evolutionary relationship between *T. gondii* RH strain HDAC3 and human HDAC1-3, as well as *Plasmodium falciparum* HDAC1, which clustered within a distinct clade (Fig. 6A). To investigate the molecular basis underlying quisinostat’s inhibitory activity, we conducted AutoDock-based molecular docking simulations. Structural analysis revealed a hydrogen bond between quisinostat and Tyr23 of *T. gondii* HDAC3, accompanied by a binding energy of −7.2 kcal/mol (Fig. 6B). This thermodynamic profile indicates a high-affinity interaction that is consistent with the observed proteomic repression of HDAC3 and its homologs. Collectively, these findings underscore the potential of quisinostat as a therapeutic agent targeting epigenetic regulation in *T. gondii* and related pathogens.

### Efficacy of quisinostat against *T. gondii* infection *in vivo*

**Fig 7:**
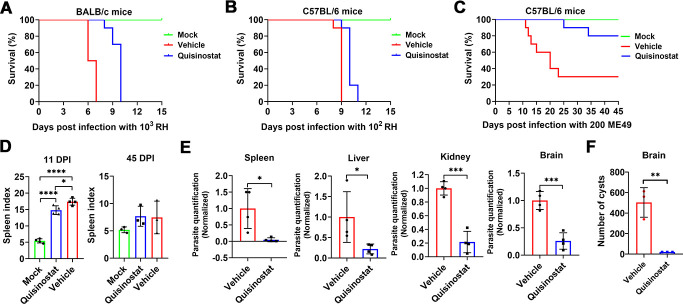
*in vivo* efficacy evaluation. (**A**) Survival curve of BALB/c mice treated with quisinostat or vehicle. Female BALB/c mice were infected with 1000T*. gondii* RH tachyzoites. After 24 h, mice were treated with quisinostat or vehicle, and survival was evaluated daily (10 mice per group). Female C57BL/6 mice were infected intraperitoneally with 100 RH tachyzoites (**B**) or 200 ME49 tachyzoites (**C**). After 24 h, mice were treated with quisinostat or vehicle, and survival was evaluated daily (10 mice per group). Meanwhile, mice in the mock group were mock infected with sterile PBS and treated with quisinostat. (**D**) Spleen index was detected at 11 DPI and 45 DPI, respectively. The spleen index was calculated as the spleen mass (mg)/mouse body mass (g). The *P* values by one-way ANOVA are indicated; *P < 0.05 and ****P < 0.0001. (**E**) Eleven days post-infection, parasite burdens in spleen, liver, kidney, and brain were determined by quantitative PCR. The *P* values by Student′s *T* test are indicated; *P < 0.05 and ***P < 0.001. (**F**) Amounts of cysts in the brains of mice after treatment with vehicle or quisinostat at 45 DPI. The *P* values by Student’s *T* test are indicated; **P < 0.01.

To determine the *in vivo* protective efficacy of quisinostat against *Toxoplasma* infection, parasite-infected mice were treated intraperitoneally with quisinostat (10 mg/kg) or vehicle. Meanwhile, mock-infected mice were treated intraperitoneally with quisinostat. All mock-infected mice treated with quisinostat showed no signs of clinical disease or weight loss (data not shown) and no mortality throughout the study. As shown in Fig. 7A, when BALB/c mice were exposed to virulent RH strain, all mice in the vehicle group died by 7 days post-infection (DPI), whereas those mice treated with quisinostat died by 10 DPI. As shown in Fig. 7B, infection with a dose of 100 RH tachyzoites resulted in all C57BL/6 mice in the vehicle group succumbing to infection by day 9, and mice treated with quisinostat all died at 11 DPI. As shown in Fig. 7C, 30% of low virulence ME49 strain-infected mice in the vehicle group survived, whereas 80% survived in the quisinostat treatment group (Fig. 7C). ME49 strain-infected mice in the vehicle group showed typical clinical signs of acute infection and started dying at 11 DPI. Meanwhile, the spleen index in the vehicle group increased significantly compared with mice in the mock group, and a significant decrease of spleen index was observed after quisinostat treatment (*P* < 0.05) (Fig. 7D). Notably, parasite burdens in different tissues (spleen, liver, kidney, and brain) were significantly decreased after quisinostat treatment compared to the vehicle group (Fig. 7E). Moreover, at 45 DPI, the spleen index decreased in survived mice, and there is no significant difference between drug-treated group and vehicle group (*P* > 0.05) (Fig. 7D). Importantly, the number of brain cysts of mice in the quisinostat-treated group was significantly decreased compared with that in the vehicle group (Fig. 7F).

## DISCUSSION

*T. gondii* harbors five HDAC homologs (TgHDAC1-5) and two subtype variants (35). Among these, TgHDAC2-4 localize to both the cytosol and nuclei (36), with TgHDAC3 specifically regulating gene expression, cell division, and bradyzoite conversion (37). However, the biological roles of other HDACs in *T. gondii* remain largely unexplored. Histone acetylation has emerged as a promising therapeutic target against *T. gondii*, yet most HDAC inhibitors (HDACis) investigated to date exhibit limited *in vivo* efficacy despite potent *in vitro* activity (17, 18). Quisinostat, a pan-HDAC inhibitor in Phase II clinical trials for cancer, demonstrates robust antimalarial activity. Our study reveals its dual efficacy against *T. gondii* both *in vitro* and *in vivo*, as evidenced by its inhibitory effects on tachyzoite proliferation and therapeutic benefits in a murine model of toxoplasmosis.

*In vitro*, quisinostat inhibited *T. gondii* tachyzoite proliferation with an EC_50_ of 25.84 pM and a selectivity index (SI) of 318.11, underscoring its high potency and low host cytotoxicity. The lytic cycle of *T. gondii*, critical for acute infection, involves invasion, intracellular replication, and egress (38). Quisinostat significantly disrupted each step of this cycle. TEM analysis revealed ultrastructural alterations including cellular swelling, membrane disintegration, and organelle degradation. These findings align with prior reports of quisinostat-induced apoptosis and cell cycle arrest in lung cancer cells (33). Here, quisinostat treatment increased LDH release and triggered apoptosis-like death in tachyzoites without altering reactive oxygen species (ROS) levels, suggesting a ROS-independent apoptotic pathway. Furthermore, quisinostat induced G1-phase cell cycle arrest, consistent with its effects in other cell types (39, 40).

Proteomic analysis identified 77 upregulated and 205 downregulated proteins in quisinostat-treated tachyzoites, distributed across nuclear, cytoplasmic, and mitochondrial compartments. GO enrichment highlighted perturbations in protein dephosphorylation and ion transport, while KEGG pathway analysis implicated non-homologous end-joining and glycine metabolism, suggesting impacts on genomic stability and energy homeostasis. As a pan-HDAC inhibitor, quisinostat downregulated HDAC2, HDAC3, and SIR2 expression. Molecular docking predicted a hydrogen bond between quisinostat and Tyr23 of TgHDAC3 (binding energy −7.2 kcal/mol), supported by phylogenetic homology between TgHDAC3 and human/malarial HDACs. These data strongly suggest that quisinostat targets HDACs to modulate epigenetic regulation in *T. gondii*.

*In vivo*, quisinostat reduced parasite burdens in multiple organs, alleviated splenomegaly, and prolonged survival in infected mice. Strikingly, cerebral cyst counts were significantly diminished, indicating potential efficacy against chronic toxoplasmosis. Parasite load, a key determinant of disease severity (41, 42), serves as a prognostic indicator for therapeutic response (43, 44). Our findings underscore quisinostat’s ability to control parasite dissemination and mitigate organ damage.

While this study establishes quisinostat’s anti-*T. gondii* activity, it has limitations. Dynamic proteomic analyzes and in-cell validation of HDAC binding are needed to confirm mechanistic specificity. Additionally, translational studies evaluating quisinostat’s pharmacokinetics and toxicity in higher animal models are essential. In summary, quisinostat exhibits potent anti-*T. gondii* activity by disrupting the lytic cycle, inducing G1 arrest, promoting apoptosis, and modulating epigenetic pathways. Its *in vivo* efficacy against both acute and chronic infection highlights its therapeutic potential. These findings provide a rationale for developing quisinostat or related HDACis as novel chemotherapeutics for toxoplasmosis.

## Data Availability

The mass spectrometry proteomics data have been deposited to the ProteomeXchange Consortium (https://proteomecentral.proteomexchange.org) via the iProX partner repository with the data set identifier PXD064903.

## SUPPLEMENTAL MATERIAL

Fig. S1 aac.01819-24-s0001.doc Determination of EC50 value of pyrimethamine against *T. gondii* RH-GFP strain*.*

Table S1 aac.01819-24-s0002.xls Proteomic profile of *T. gondii* RH tachyzoites after quisinostat treatment*.*

ASM does not own the copyrights to Supplemental Material that may be linked to, or accessed through, an article. The authors have granted ASM a non-exclusive, world-wide license to publish the Supplemental Material files. Please contact the corresponding author directly for reuse.
