# Supplementary figures and images for: In vitro and in vivo activity of quisinostat against Toxoplasma gondii
Source: Antimicrob Agents Chemother. 2025 Jul 31;69(9):e01819-24. doi: 10.1128/aac.01819-24 (PMC12406659; doi:10.1128/aac.01819-24)

**
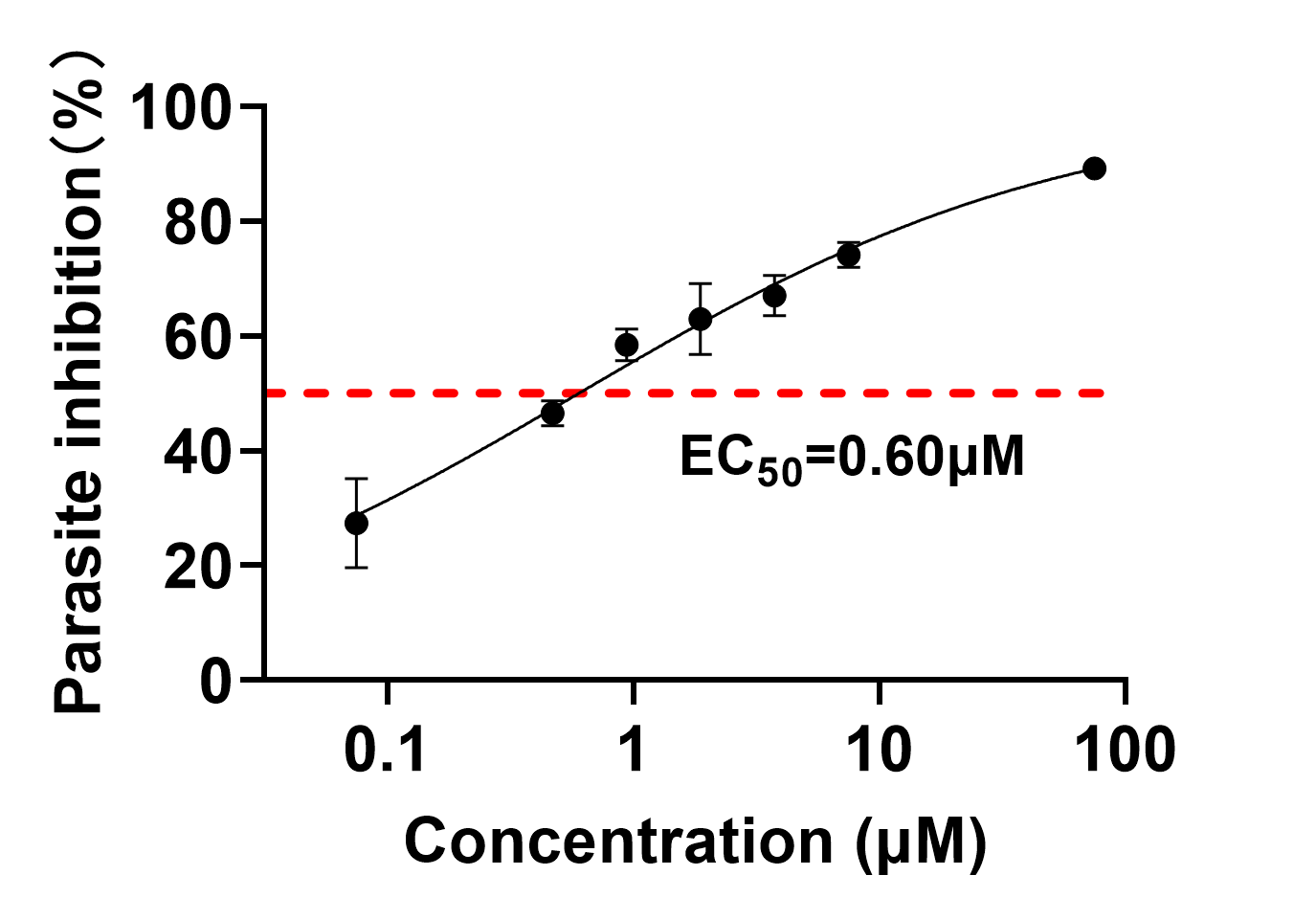
**

**FIG S1**. Determination of EC50 value of pyrimethamine against *T. gondii* RH-GFP strain.

Supplement: Fig. S1 — Determination of EC50 value of pyrimethamine against T. gondii RH-GFP strain. [file aac.01819-24-s0001.doc]
